# Large-Scale Avian Influenza Surveillance in Wild Birds throughout the United States

**DOI:** 10.1371/journal.pone.0104360

**Published:** 2014-08-12

**Authors:** Sarah N. Bevins, Kerri Pedersen, Mark W. Lutman, John A. Baroch, Brandon S. Schmit, Dennis Kohler, Thomas Gidlewski, Dale L. Nolte, Seth R. Swafford, Thomas J. DeLiberto

**Affiliations:** 1 USDA/APHIS/Wildlife Services National Wildlife Disease Program, Fort Collins, Colorado, United States of America; 2 USDA/APHIS/Wildlife Services National Wildlife Research Center, Fort Collins, Colorado, United States of America; 3 USDA/APHIS/Wildlife Services National Feral Swine Damage Management Program, Fort Collins, Colorado, United States of America; 4 USDOI United States Fish and Wildlife Service, Yazoo City, Mississippi, United States of America; Iowa State University, College of Veterinary Medicine, United States of America

## Abstract

Avian influenza is a viral disease that primarily infects wild and domestic birds, but it also can be transmitted to a variety of mammals. In 2006, the United States of America Departments of Agriculture and Interior designed a large-scale, interagency surveillance effort that sought to determine if highly pathogenic avian influenza viruses were present in wild bird populations within the United States of America. This program, combined with the Canadian and Mexican surveillance programs, represented the largest, coordinated wildlife disease surveillance program ever implemented. Here we analyze data from 197,885 samples that were collected from over 200 wild bird species. While the initial motivation for surveillance focused on highly pathogenic avian influenza, the scale of the data provided unprecedented information on the ecology of avian influenza viruses in the United States, avian influenza virus host associations, and avian influenza prevalence in wild birds over time. Ultimately, significant advances in our knowledge of avian influenza will depend on both large-scale surveillance efforts and on focused research studies.

## Introduction

In 2006, the United States of America (USA) Departments of Agriculture (USDA) and Interior (DOI), along with multiple state and tribal agencies, implemented a nationally coordinated, avian influenza surveillance effort in wild birds. This surveillance effort was initially motivated by concern stemming from the involvement of wild birds in novel outbreaks of highly pathogenic avian influenza virus (HPAIV) in Asia during 2004 and 2005 [1]–[4]. This strategy was based on the premise that while the greatest risk of HPAIV introduction was from the illegal importation of poultry or poultry products, as well as through the illegal trade of wild and exotic birds, HPAIV could also be introduced through wild bird migration [4]–[6]. Although several long-term research projects studying avian influenza viruses (AIV) in wild birds have been conducted [7]–[9], no coordinated early detection system existed that could rapidly identify the introduction or emergence of HPAIV in wild birds. An ancillary benefit of this large-scale surveillance system was an unprecedented amount of data on all AIVs, not simply HPAIVs, in wild birds. Since there was limited knowledge on how H5N1 would affect North American wild birds at the onset of the survey, the surveillance system was designed to maximize the chance of detecting H5N1 regardless of clinical characteristics, species involved, or geographic location [1], [2]. In essence, the surveillance system was designed to detect as many AIVs as possible, regardless of their pathogenicity.

Avian influenza viruses have a worldwide distribution in both wild and domestic birds and they are often divided into two groups, low pathogenic AIVs and high pathogenic AIVs. The pathogenicity designation is based on criteria set forth by the World Organization for Animal Health (OIE) and these criteria include amino acid sequence, the lethality of the virus when inoculated into four to six week old specific pathogen-free chickens, or having an intravenous pathogenicity index >1.2 [10]. Avian influenza virus classification is based on one of the 18 hemagglutinin (HA) and 11 neuraminidase (NA) surface proteins [11]; most HA/NA combinations have been isolated from avian species, but HPAIVs have only been found in H5 or H7 viruses. Highly pathogenic avian influenzas can cause up to 100% mortality in infected domestic poultry and while there are occasionally die-offs in non-domestic birds as well, infections can often be asymptomatic in these wild species. Waterfowl in particular have been documented as having the highest general AIV prevalence rates, as well as the greatest subtype variety [12]. The ability of waterfowl to cover large geographic distances when migrating, combined with the substantial prevalence and diversity of AIVs they can carry, offers the opportunity for novel AIVs to emerge through co-infection events, as well as through the introduction of AIVs from different regions into immunologically naïve populations. These events represent a threat to the domestic poultry industry, as well as to human populations in the case of AIVs that are transmissible to people.

The surveillance strategy was designed to provide a conceptual framework that combined risk assessment methods with traditional surveillance designs for early detection and response in the case of pathogen introduction. The objectives were to identify the necessary components of influenza surveillance in order to efficiently and effectively conduct large-scale disease surveillance in wild birds. This effort solicited expert opinion from the USDA Wildlife Services, the United States Geological Survey, the United States Fish and Wildlife Service, the Centers for Disease Control and Prevention, the Association of Fish and Wildlife Agencies, and National Flyway Councils. Surveillance targeted live wild birds (as well as birds involved in morbidity/mortality events) that had the highest risk of being exposed to, or infected with, HPAIV because of their migratory movement patterns [13]–[15]. This included bird species that migrated directly between Asia or Europe and the USA, birds that may have been in contact with species from areas with reported AIV outbreaks, or species that were known reservoirs of AIV [13], [16]–[18].

The early detection system was implemented on 1 April 2006 and continued through 31 March 2011. This paper summarizes the resultant data, with the objectives being, (1) to provide an overview of the USA's wild bird early detection system for HPAIV, (2) to report specific results associated with that effort, (3) to identify wild bird AIV hotspots within the continental USA that could aid in future, targeted surveillance efforts and (4), to highlight patterns of infection that can be revealed through large-scale pathogen surveillance efforts in wildlife.

## Methods

### Ethics Statement

Wildlife surveillance activities were carried out in accordance with permitting agencies and, if applicable, with the permission of private landowners. Migratory bird capture and sampling were approved by the USA Fish and Wildlife Service (Permit Number MB124992) for HPAI surveillance. Samples collected at hunter-check stations were collected through state and local officials and with the permission of participating hunters.

### Surveillance

The surveillance system utilized a risk-based approach to conduct wild bird surveillance [1]. The wild bird metacommunity was stratified by flyway and then again by species, both to take into account their potential role in moving HPAIV into the USA and to reflect the severity of HPAIV infection that is associated with different species [16]. Sampling strata were further refined to prioritize areas and species within flyways. Flyway councils, which are administrative organizations with one member from each state and province located within that flyway, also contributed to prioritizing areas and species for surveillance. The species of interest varied by location, time of year, and sampling method employed (i.e. samples from hunter-harvest birds do not cover all species because of hunting regulations). Additional sampling criteria included, 1) historic disease prevalence, 2) species-specific migratory pathways, 3) geographic size and location of each state, 4) wetland habitat and location in relation to shoreline, 5) waterfowl expert input from the Flyway Councils and the Association of Fish & Wildlife Agencies, and 6) bird-band recovery data. Agencies were requested to sample individuals/states from the flyway priority list until state-wide target numbers were achieved. Agencies within each state decided which species from the flyway priority list to sample.

Strategies employed for collecting surveillance data on AIVs in wild birds were 1) investigation of morbidity and mortality events, 2) hunter-harvest surveillance, 3) live-bird sampling, 4) sample collection from sentinel species, and 5) environmental fecal sampling. Each strategy had biological, logistical, and economic benefits and constraints [1], [2]; consequently, agencies chose strategies that would be most effective in their state depending on specific sampling locations and times of year, but the vast majority (mean = 98.4%) of samples were collected from live-capture or hunter-killed birds. Samples from morbidity and mortality events were taken from a subset of individuals that represented each species involved in the event to determine if an AIV was associated with morbidity or mortality.

Standardized protocols were implemented by all agencies to collect, ship, and test wild bird samples for AIV [2], [19]. The surveillance protocol purposely collected both an oropharyngeal swab and a cloacal swab in order to increase the chance of virus detection and to maximize the amount of AIV data generated [6], [20], [21]. Both swabs were added to a single cryovial containing brain-heart infusion (BHI) media and this represented one sample for one bird (i.e. the number of birds sampled and the number of samples collected are used interchangeably).

Bird samples were screened at one of 43 participating National Animal Health Laboratory Network (NAHLN) facilities. This laboratory network is certified by the USDA/APHIS, National Veterinary Services Laboratories (NVSL). The OIE (World Organization for Animal Health) has certified the NVSL as the reference laboratory for AIV diagnostics in the USA. Detailed descriptions of diagnostic testing protocols have been described previously [1], [2]. Briefly, wild bird samples were tested at a NAHLN facility for AIV by rRT-PCR using the matrix (M) gene primer [22]; if positive, samples were tested for H5 and H7 by rRT-PCR [22]. The H7-specific assay was modified in 2008 when a new reference test was developed [23]. Positive H5 or H7 rRT-PCR samples were express shipped to the NVSL within 24 hours of a presumptive finding for virus isolation and pathogenicity testing [2]. Samples that were negative for H5 and H7 were shipped to the USDA National Wildlife Disease Program, Wild Bird Tissue Archive in Fort Collins, CO, USA. Specific rRT-PCR assays, virus isolation, subtyping, and pathogenicity tests were performed according to international guidelines [3], [24], [25].

### Analyses

Surveillance results were available in real-time in order to detect the introduction of HPAIVs, but data presented here were also retrospectively analyzed and mapped using ArcMap v. 10.0. The global Moran's I statistic was used to examine underlying spatial autocorrelation patterns in AIV M positive data, using the number of positive birds detected at the county level. Distance band results from the Moran's I statistic were then incorporated into the Getis-Ord Gi* statistic to identify the specific locations where high and low values of AIV M positive samples were clustered (Z scores, 95% confidence levels (CI) +1.96 and −1.96 standard deviations). These analyses excluded samples collected outside of the 48 contiguous states in order to eliminate non-logical clustering.

Wild bird samples were analyzed using the Cochran-Armitage trend statistic to determine if changes in apparent prevalence occurred during the course of surveillance. This analysis was performed on M gene positive prevalence in both hatch-year birds and in after hatch-year birds, because of previously reported differences in AIV prevalence by age group [7], [12]. Collection year (based on a biological year (BY) running from 1 April to 31 March the following year) and avian functional group data were analyzed using logistic regression, with results of the AIV M assay for each sample as the binary dependent variable. Avian functional groups include dabbling ducks (mallards and other species that feed under the surface of the water by tipping), diving ducks (ducks which feed by diving under the surface of the water), geese and swans, gulls and terns, perching ducks (ducks that perch in trees), shorebirds, and other species. The relationship between avian functional group sampling by collection year was examined using chi-square analysis, with correspondence analysis used to determine relative contributions to the chi-square statistic. Goodness of fit tests determined if M positive AIV infections were equally distributed among avian functional groups based on proportion of samples collected.

Chi-square analysis evaluated if population increases occurred over sampling years for the three dabbling duck species that were most commonly sampled and were most commonly AIV M positive. The number of days it took to receive samples at the lab after collection and the number of days it took to test samples after they arrived at the lab were analyzed over time using a generalized linear model with a Poisson distribution and a log link function for count data. This was done to determine if sample handling and processing became increasingly efficient over time, which could have the potential to bias results. All statistical analyses were run in SAS, version 9.2.

## Results

**Figure 1:**
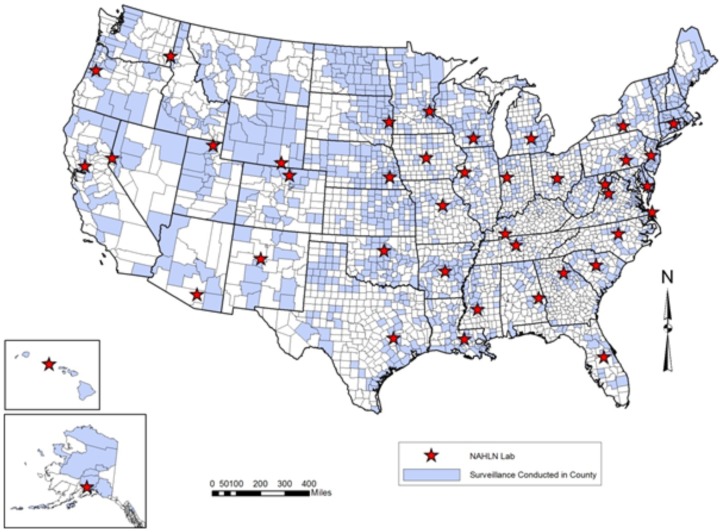
Hunter-harvested and live wild birds were sampled for avian influenza virus throughout the USA. Samples were collected in shaded counties and testing occurred at starred NALHN laboratory locations.

**Figure 2:**
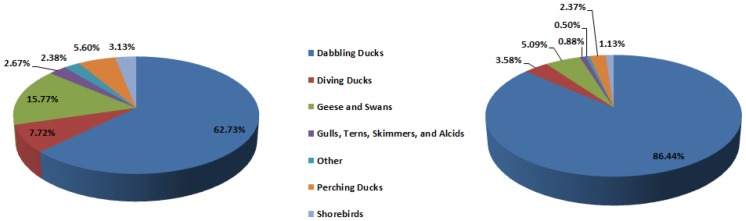
Percentage of samples collected for wild bird influenza virus surveillance by avian functional group (a); and the proportion of samples by avian functional group (b) that were influenza virus positive by M gene rRT-PCR (n = 197,885).

Over the five-year surveillance effort, the USDA coordinated the collection of samples from 283,434 wild birds that originated from more than 250 bird species. Approximately 98% of wild bird samples collected were from hunter–harvest/agency-harvest or live wild birds. Remaining samples were either morbidity/mortality events (0.09%) or sentinel species (1.51%). These samples were collected from locations throughout the USA including Alaska, Hawaii, and multiple territories of the USA (Figure 1). Samples from dabbling ducks, geese and swans, and diving ducks accounted for 86.2% of all samples collected (Figure 2a). This pattern was consistent across all flyways except for Oceania where only 2,262 samples were collected by the USDA, the majority of which were from wading birds, passerines, and other species. None of the 283,434 samples tested positive for HPAIV.

**Figure 3:**
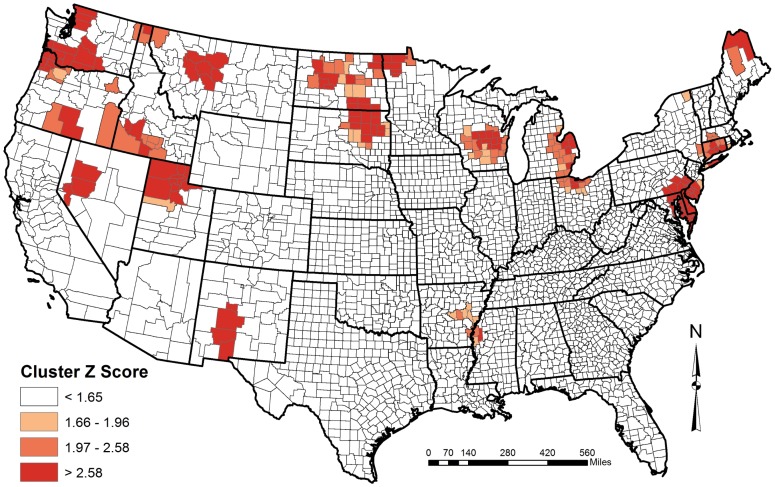
Continental scale map showing spatial clustering hot spots of avian influenza virus M gene positive wild birds. Z-score results from the Getis-Ord Gi* analyses: >1.65 = 90% significant, 1.66–1.96 = 90%–95% significant, 1.97–2.58 = 95%–99% significant, >2.58 = 99% significant.

**Figure 4:**
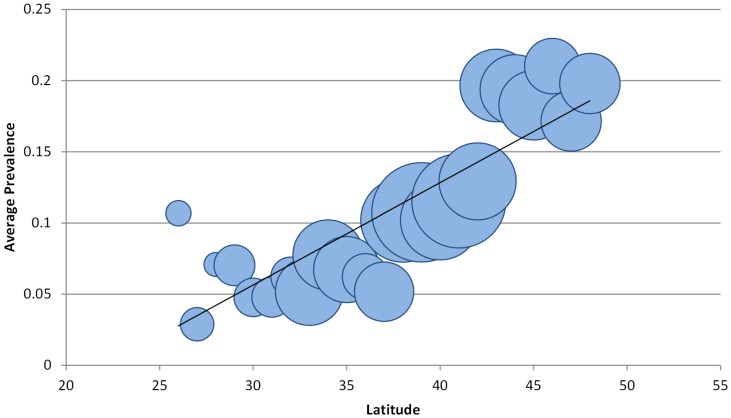
Average prevalence of M gene rRT-PCR positive samples plotted against latitudinal degree of collection site. Circle diameter represents samples size at each latitudinal degree.

Analyses reported here encompass data (n = 197,885) collected between 2007 and 2011. The Moran's I statistic indicated that high and low values of AIV M positive birds were more spatially clustered than would be expected if underlying processes were random. A peak in z-score values was seen at a 100,000 meters (z-score = 19.73, p<0.0001), which was then used as the distance band in the Getis-Ord Gi* hotspot analysis. Resulting z-scores revealed patterns similar to the clustering seen in the Moran's I results, with significantly different clustering of positive and negative AIV M positive wild birds (Figure 3). Hot spots were primarily seen in the northern latitudes of the continental USA, and were primarily located in known staging areas for large numbers of migratory birds, such as Delaware Bay and the Prairie Pothole Region in the northern Great Plains. Reinforcing these findings is the pattern of higher average M positive infections in the northern latitudes when data are analyzed by latitudinal degree (Figure 4).

Mean apparent prevalence of AIVs in wild birds collected between 1 April 2007–31 March 2011 was 11.4% (95% CI 11.3–11.6). Prevalence was highest in dabbling ducks (mean prevalence = 15.8%, 95% CI 15.6–16.0), and this functional group accounted for 86.4% (19,582/22,654) of AIVs detected, a disproportionately high number (χ^2^ = 5930, P<0.001) when compared to the percentage of samples collected from dabbling ducks (62.73%, 124,127/197,885); all other functional groups accounted for only 13.55% (3,072/22,654) of AIV M positives based on the rRT-PCR M assay conducted at NAHLN facilities (Figure 2b). Wild bird samples were collected during every month of the year, but a majority of samples, 83% (164,656/197,885), were collected from August through January of each year to correspond with known seasonal increases in wild bird AIV infection [26], [27]. This increase in AIV positive birds is thought to be associated with virus exposure in new, immunologically naïve hatch-year birds and correspondingly, the number of AIV M positive wild birds began to increase each year in July, reached a peak in October, and then began a gradual decline [28].

**Figure 5:**
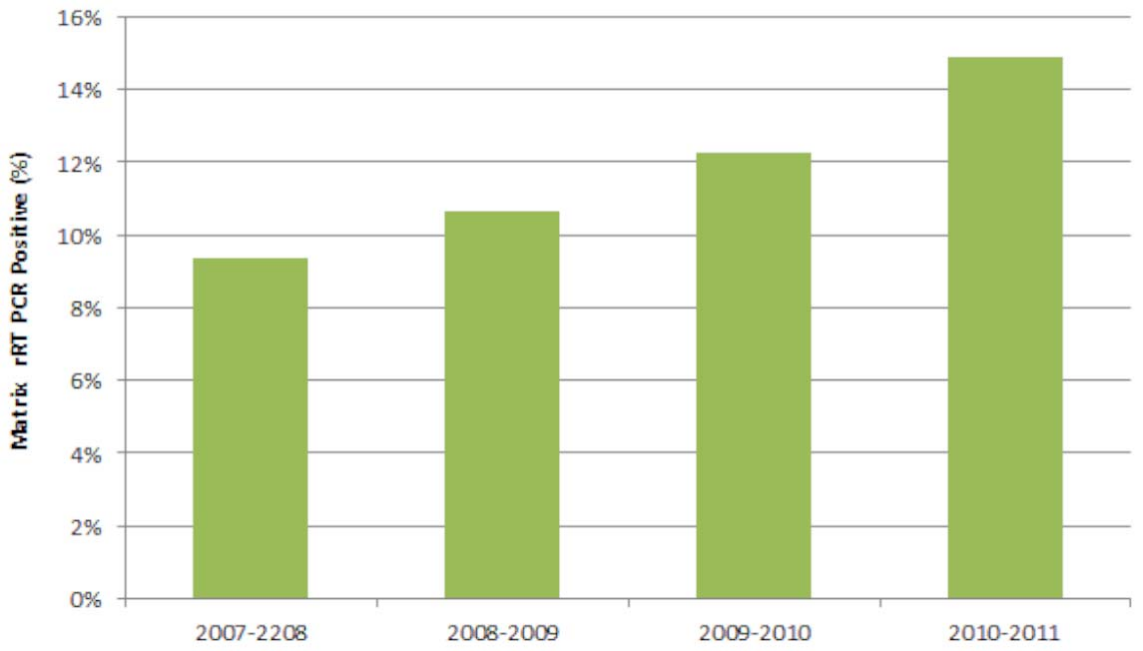
Influenza virus M gene rRT-PCR positive wild bird samples. Average prevalence and sample size are plotted by year.

The mean prevalence of AIV M positive wild birds increased each year (Cochran-Armitage test statistic = −24.4, p<0.0001, Figure 5). This increase occurred even though the number of birds sampled per year decreased over time as a result of reduced funding. This trend of increasing prevalence over time was consistent in both hatch-year birds (Cochran-Armitage test statistic = −13.14, p<0.0001) and after hatch-year birds (Cochrane-Armitage test statistic = −15.97, p<0.0001). When examining M positive results by functional group and year, the only functional group that showed a consistent increase in M positive birds over time was dabbling ducks (Odds ratios: BY07 versus BY08 = 0.89, (0.86–0.93), BY08 versus BY09 = 0.87 (0.84–0.91), and BY09 versus BY10 = 0.69 (0.66–0.72). Dabbling ducks were the primary functional group sampled, because of previously reported associations with AIV and because they are known to harbor the virus without exhibiting clinical signs. While overall sampling by functional group significantly differed from year to year (χ^2^ = 2003.7, p<0.0001), the percentage of samples belonging to dabbling ducks remained relatively consistent across time (% of total samples that came from dabbling ducks: BY07 = 62.04, BY08 = 61.44, BY09 = 65.43, and BY10 = 62.93), with only BY09 exhibiting a higher sample size (P<0.0001). Correspondence analysis demonstrated that variation in dabbling duck sampling over time contributed less to the significant chi-square association (3.68%) than any other functional group. Neither the three dabbling duck species that had the highest AIV prevalence (Mallards, Blue-winged Teal, American Black Duck) in this study (χ^2^ = 0.02, p<0.99) nor the three dabbling duck species that had the highest sample numbers (Mallard, Blue-winged Teal, Northern Shoveler) from this study (χ^2^ = 0.001, p<0.99) had statistically significant population level increases during this AIV sampling effort that could have contributed to the observed trend in AIV prevalence.

The time it took samples to arrive at the laboratory after collection was analyzed to determine if the increase in M positive wild birds was a result of higher quality samples being submitted (i.e. less sample degradation) over the course of the surveillance effort. Neither the time period between collection and arrival at diagnostic labs (mean in number of days: BY07 = 1.73 (SE = 0.006), BY08 = 1.79 (SE = 0.006), BY09 = 1.46 (SE = (0.008), BY10 = 1.48 (SE = 0.01)) nor the time between sample arrival at a lab and testing (mean in number of days: BY07 = 4.1(SE = 0.008), BY08 = 3.8 (SE = 0.008), BY09 = 4.0 (SE = 0.01), BY10 = 4.3(SE = 0.01)) demonstrated a marked change over time.

There were 2,521 samples collected and submitted for testing that were H5 (n = 2,130) or H7 (n = 391) gene positive by rRT-PCR at a NAHLN laboratory. Once again dabbling ducks accounted for a disproportionate majority of H5 (1,949/2,130 = 91.5%) and H7 (351/391 = 89.7%) positive samples (χ^2^ =  790.2, P<0.0001). Out of all M positive samples, H5 and H7 positives (based on rRT-PCR) represented 9.3% (2,130/22,654) and 1.7% (391/22,654), respectively. The apparent prevalence of H5 positive wild birds also increased from BY07 through BY10 (Cochran-Armitage test statistic z = −10.64, p<0.0001). H7 positive wild bird trends were not analyzed because of limited data.

Initial virus isolation yielded 513 AIV isolates where both the HA and NA proteins could be isolated and out of these isolates, 43 unique combinations of HA and NA were positively identified (Table 1). Mixed infections, consisting of simultaneous infection with multiple viruses were also identified in 20 additional samples, but the exact HA/NA combination could not be conclusively determined. The most common HA groups identified were H3, H4, H5, and H7. This is not surprising since this surveillance effort targeted the identification of H5 and H7 viruses. The most common NA groups were N2, N3, and N6. The most common viruses identified were H5N2, H4N6, and H7N3, comprising 25.3% (130/513), 9.7% (50/513), and 17.9% (92/513) of the AIV subtypes identified, respectively (Table 1).

**Table 1:** Virus isolation yielded 513 AIV isolates consisting of 43 different HA/NA combinations, although exact HA/NA combinations could only be determined for 413 isolates*.

	H1	H2	H3	H4	H5	H6	H7	H8	H9	H10	H11
**N1**	7		7		8	6	3				
**N2**	2	1	20	20	130	9	3			2	1
**N3**		4	1		19		92			1	
**N4**						3	2	1			
**N5**			1		3		1				
**N6**			10	50	2		5				
**N7**					1		8			10	
**N8**			19	13	1	1	1			3	
**N9**			2	1	3		6				10

* Mixed infections, consisting of simultaneous infection with multiple viruses were also identified in 20 additional samples, but the exact HA/NA combination could not be determined.

## Discussion

Data presented here resulted from a nationally coordinated HPAIV early detection system in wild birds and provide valuable insight into the ecology of low pathogenic AIVs in waterfowl at an unprecedented scale. As documented in previous studies, the vast majority of AIVs were detected in dabbling ducks. While the USDA effort used a targeted approach resulting in a majority of the samples coming from dabbling ducks, AIV prevalence in this functional group was disproportionally high (86.4%). The majority of H5 (91.5%) and H7 (89.7%) AIVs detected were also detected in dabbling ducks. These results reinforce the important role of dabbling ducks as a natural reservoir of AIVs, especially for these viruses which have the potential to re-assort into novel AIVs that can have differing host affinities, transmission efficiencies, and virulence.

Using this unique dataset, spatial analyses carried out on sampling within the continental USA revealed regions with high numbers of AIV M positive wild birds. Some of these findings are corroborated by previous research that has documented AIV activity in similar areas [8], [29], [30]. Clusters of AIV M positive birds were primarily concentrated in the northern latitudes of the USA and in wetland areas or specific water bodies that offer migrating waterfowl stopover points on the landscape, including Delaware Bay on the Eastern Seaboard, Stillwater and Carson Lake Wetlands in Nevada, and the Prairie Pothole Region in the northern Great Plains. The only clusters located entirely below the 40° parallel were along a riparian area of the Rio Grande River in New Mexico (Figure 4), where researchers have suggested that the limited amount of water on the landscape leads to a bottlenecking of migratory water birds in this region [31], and one along the Mississippi River flyway in Arkansas and Mississippi (Figure 4). The association of M positive wild bird clusters with biologically relevant landscape features suggests that future low pathogenic AIV surveillance efforts can be efficiently implemented by preferentially targeting sample collection in these areas. Cluster analysis results are reinforced by descriptive data demonstrating that the prevalence of M positive birds increases with latitude (Figure 4). While this pattern is likely influenced by multiple variables, the general trend reveals areas of high activity that could be used to implement future surveillance and management efforts. These northern latitude data primarily encompassed birds during migration (September through March), when they are likely interacting with large numbers of birds of multiple species, offering the opportunity for influenza transmission and circulation of multiple subtypes [28], [32]. These migratory stopover points would also include many new hatch-year birds, which represent a new crop of susceptible individuals to fuel transmission [7], [12], [28], [33].

Annual prevalence of AIVs in wild birds throughout this effort varied within ranges reported in previous studies; however, to our knowledge, this is the first time statistically increasing trends in AIVs in wild birds have been documented on such a large spatial scale. A similar trend was observed for prevalence of H5 viruses, suggesting that the increase in M positive wild birds could at least be partially attributed to an increase in H5 occurrence; however, since H5 viruses only accounted for 9.3% of M positive birds, other subtypes likely played a role as well. Data from the DOI Breeding Bird Survey [34] on the three dabbling duck species with the highest AIV prevalence in this survey and on the three dabbling duck species with the highest sample numbers collected during this survey, revealed that their populations did not significantly increase in North America from 2007–2011 (Mallards, Blue-winged Teal, American Black Duck; and Mallard, Blue-winged Teal, Northern Shoveler, respectively (p = 0.9994)). Therefore, the increase in prevalence was not simply reflecting an increase in the most prolific dabbling duck species. The number of dabbling ducks sampled was relatively consistent over the course of this study, with only one year (BY2009) having slightly more dabbling ducks sampled when compared to all other sample years. Therefore, the increase in AIV prevalence over time was not an artifact of sampling an increasing number of dabbling ducks over time.

The geographic and temporal scope of this effort provided an opportunity to document an increasing trend in AIV prevalence at biologically relevant regional (flyway) and continental scales. It also provides evidence that this trend did not result from differences in population size, sample size, sampling efficiency, or diagnostic testing efficiency. The actual mechanism and implication for the observed trend is unknown. The increasing prevalence may represent part of a multi-year cycle of AIVs in their natural reservoirs [35].

The most commonly isolated hemagglutinin groups were H3, H4, H5, and H7. Prior to this effort, conventional wisdom suggested that H3, H4, and H6 viruses were the most common hemagglutinin types circulating in dabbling ducks and that H5 and H7 viruses were infrequently or sporadically isolated [12], [36], [37]. Results reported here targeted the identification of H5 and H7 viruses (only samples that were H5 or H7 gene positive by rRT-PCR were sent for subtyping and pathogenicity testing) and therefore the data are biased, but they do suggest that in addition to H4 viruses, H5 and H7 viruses were commonly in circulation during the surveillance effort. Previous research suggested that H5N2 viruses were becoming more prevalent in wild ducks [9], [19] and this was also the most common virus isolated in this study. A diverse array of low pathogenic AIV isolates from HA groups other than H5 and H7 were detected throughout the study, including multiple H10 and H11 viruses. While our testing protocols preferentially targeted H5 and H7 AIVs, isolation of other HA groups was likely a result of mixed infections. This would occur when a bird is co-infected with multiple AIVs, but only one of those viruses grows during isolation because of competitive advantage. Another possibility that would lead to isolation of non H5 or H7 HAs from H5 or H7 rRT-PCR positive samples is that the genetic material detected by rRT-PCR was not from live virus. In this case, live virus from other AIV strains would have grown during virus isolation.

Future studies should focus on identifying any potential relationships of these subtypes among wild birds and poultry. During the course of this effort, H5N2, H4N6, and H7N3 viruses were responsible for a number of AIV outbreaks in USA poultry [38]–[40]. Although more detailed genetic analyses are still pending, the occurrence of these viruses in wild birds suggests a possible link between AIV in wild birds and poultry. Further analysis of these trends will be difficult since data collected ceased in 2011 when the national surveillance program was discontinued; however, future research could examine if the wild bird AIV patterns reported here are associated with downstream changes in AIV infections in poultry and other species. Using data collected during the course of this surveillance program to refine future efforts should enhance efficiency and also allows for targeted sampling to answer these and other pressing research questions.

The early detection system was specifically designed to detect HPAIV. No HPAIVs were detected in the USA wild bird population. The majority of samples collected through this effort were archived at the USDA National Wild Bird Tissue Archive located at the Colorado State University Diagnostic Laboratory in Fort Collins, Colorado. This archive has proven an invaluable resource for increasing knowledge of AIVs in their natural reservoir. Researchers from the National Wildlife Research Center and a number of universities continue to conduct virus isolation and genetic sequencing on samples stored at the archive in order to provide new insights on AIVs in wild birds.

Prior to 2005 much of our knowledge of AIVs in wild birds came from research studies designed to examine the ecology of viruses at specific locations, times, and in a limited number of species. Such small-scale studies performed in focused geographic ranges over relatively short periods of time are critical for understanding host–virus relationships; however, extrapolating this knowledge to metapopulations and metacommunities of wild birds at regional and continental scales can be problematic. Large-scale surveillance programs such as this one in the USA and others [7], [37], [41] are important for providing ecological data on infections at politically and biologically relevant scales, which can be used to establish infection status in target populations [42]. These programs also allow for modeling disease spread and more precise risk analyses [32]. Large-scale surveillance projects such as the one undertaken in this effort will improve our understanding of the ecological parameters involved in the maintenance and transfer of AIVs from natural reservoirs to humans [12].
